# RNA binding activates RIG-I by releasing an autorepressed signaling domain

**DOI:** 10.1126/sciadv.aax3641

**Published:** 2019-10-02

**Authors:** T. H. Dickey, B. Song, A. M. Pyle

**Affiliations:** 1 Department of Molecular, Cellular and Developmental Biology, Yale University, New Haven, CT, USA.; 2 Howard Hughes Medical Institute, Yale University, New Haven, CT, USA.

## Abstract

The retinoic acid–inducible gene I (RIG-I) innate immune receptor is an important immunotherapeutic target, but we lack approaches for monitoring the physical basis for its activation in vitro. This gap in our understanding has led to confusion about mechanisms of RIG-I activation and difficulty discovering agonists and antagonists. We therefore created a novel fluorescence resonance energy transfer–based method for measuring RIG-I activation in vitro using dual site-specific fluorescent labeling of the protein. This approach enables us to measure the conformational change that releases the signaling domain during the first step of RIG-I activation, making it possible to understand the role of stimulatory ligands. We have found that RNA alone is sufficient to eject the signaling domain, ejection is reversible, and adenosine triphosphate plays but a minor role in this process. These findings help explain RIG-I dysfunction in autoimmune disease, and they inform the design of therapeutics targeting RIG-I.

## Teaser

Direct monitoring of RIG-I activation by FRET reveals reversible activation by RNA alone.

## INTRODUCTION

Retinoic acid–inducible gene I (RIG-I) is a human pattern recognition receptor (PRR) that is a promising therapeutic target (*1*). Like most PRRs, its activation leads to a potent immune response that may be useful if harnessed for immunooncology, vaccination, or antiviral therapy (*2*–*4*). Conversely, inappropriate activation of RIG-I can lead to autoimmune disorders (*5*). The pathogen-associated molecular pattern (PAMP) recognized by RIG-I has been identified as a 5′-triphosphorylated blunt duplex RNA (*6*). However, many less-effective ligands have also been reported due to the complexity of the cellular immune response (*7*). PAMPs can have cell type–specific activity, cross-talk with other PRRs, contain impurities, or fall prey to a number of other caveats that produce misleading results in cell culture. Careful in vitro investigation of PRRs and their ligands is necessary to complement cell-based results and has led to notable advances in our understanding of RIG-I (*8*, *9*).

Many of the advances in our understanding of RIG-I have come from in vitro structural work. This includes structures of RIG-I bound to a minimal RNA ligand, confirming its recognition of a 5′-triphosphorylated blunt duplex RNA (*10*, *11*). The apo structure of RIG-I has been of particular interest because it revealed an autoinhibited conformation where the critical signaling domain (a pair of caspase recruitment domains, known as 2CARD) forms an interaction interface with the hel2i domain, effectively tucking it into the body of the protein (*12*). Upon activation of RIG-I, 2CARD is known to interact with downstream proteins (such as MAVS) to transmit the immunostimulatory signal (*13*–*17*). Therefore, it was predicted that the 2CARD/hel2i contact is broken upon RIG-I activation and that 2CARD is flipped into solution, making it sterically accessible for interaction with other proteins (*12*).

In vitro biochemical work largely agrees with the structural model for RIG-I activation, but there are issues that stem from shortcomings in available methodologies. Traditional RNA binding and adenosine triphosphatase (ATPase) techniques have been applied to RIG-I with great success (*18*–*22*). However, there are many steps in the RIG-I signaling cascade between these commonly measured in vitro activities and the signaling outputs that are measured in cell culture experiments. This has led to some inconsistencies between results from RNA binding, adenosine triphosphate (ATP) hydrolysis, and cell signaling experiments. For example, RNAs that bind RIG-I or stimulate ATP hydrolysis do not always signal in cells (*18*, *20*). Inconsistencies between these results hinder the identification and validation of proposed agonists and antagonists of RIG-I. In addition, these inconsistencies prevent a complete understanding of RIG-I function and its response to the binding of specific ligands. For example, ATP has been proposed to be involved in RIG-I proofreading, conformational rearrangement, translocation, and multimerization, and all these processes have been suggested to affect signaling by the protein (*18*, *23*–*25*). However, the physical act of signal presentation by RIG-I (2CARD ejection) has not been directly measured and therefore cannot be differentiated from the many downstream events required for signal transduction.

2CARD ejection is necessarily the first step in RIG-I activation, but existing methods for measuring this conformational change have limitations. Hydrogen/deuterium exchange (HDX) and small-angle x-ray scattering (SAXS) have been used to monitor the relative position of 2CARD, but these methods require artificially high concentrations of reagents and have limited throughput (*26*–*28*). In addition, HDX reports subtle conformational changes that alter solvent accessibility, and one cannot distinguish these from large conformational changes. SAXS is sensitive to large conformational changes but is limited by its low resolution.

We set out to bridge the methodological gap between existing in vitro biochemical experiments and cell culture experiments. These efforts led to the creation of a validated assay for RIG-I activation using fluorescence resonance energy transfer (FRET). This FRET assay measures 2CARD ejection, which better represents the first stages of RIG-I activation than other in vitro techniques and is more readily controlled than cell-based techniques. The quantitative and facile readout of the assay also provides critical advantages over existing methods for monitoring the position of the 2CARD domain. Here, we have used the FRET assay to answer questions about the relative contributions of RNA and ATP to RIG-I activation.

## RESULTS

### Design of a FRET reporter for RIG-I activation

**Fig. 1:**
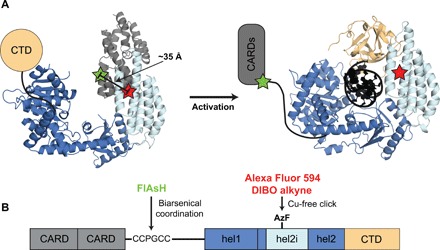
Design of a FRET reporter for RIG-I activation. (**A**) Donor and acceptor fluorophores were placed on the 2CARD and hel2i domains of RIG-I, respectively. Models of RIG-I in the autoinhibited state [Protein Data Bank (PDB) ID: 4A2W] and the activated state (PDB ID: 5F9H) predict that the distance between fluorophores changes significantly upon activation. (**B**) FlAsH donor fluorophore was attached by inserting a tetracysteine motif (CCPGCC) immediately C-terminal to the 2CARD domain. Alexa Fluor 594 acceptor fluorophore was attached via copper-free click chemistry to the unnatural amino acid azido-phenylalanine (AzF), which was incorporated by amber codon suppression. Structures were modeled using PyMOL (*49*).

We designed a reporter for RIG-I activation that measures the position of the 2CARD domain relative to the helicase core (Fig. 1A). 2CARD directly contacts the hel2i domain in the autoinhibited state, but 2CARD is predicted to be moved significantly further away in the active conformation, allowing it to interact with downstream partners. While a complete structure of active RIG-I containing the 2CARD domain is lacking, we know that 2CARD is separated from hel2i by 275 amino acids, including a flexible 50–amino acid linker between 2CARD and the helicase core. Thus, RIG-I activation should lead to a change in distance between the 2CARD and hel2i domains that is both functionally relevant to signaling and large enough to be measured by FRET.

The design of the FRET construct involves incorporation of small organic fluorophores unlikely to perturb RIG-I function, and it requires the insertion of chemically orthogonal tags at internal positions of the protein. We FlAsH-labeled 2CARD by inserting a small tetracysteine motif (CCPGCC) in the disordered and poorly conserved linker immediately C-terminal to 2CARD (Fig. 1 and fig. S1). This tetracysteine motif specifically coordinates the biarsenical fluorescein derivative FlAsH (*29*). The location of the tetracysteine tag was selected because it is solvent accessible, insertion of the tag is unlikely to perturb RIG-I function, and the distance to hel2i is minimal in the autoinhibited state. For attachment of the second fluorophore, we mutated amino acid Glu^494^ in the hel2i domain to the unnatural amino acid azido-phenylalanine (AzF). This azide functionality allows specific labeling with alkyne-containing fluorophores via click chemistry. Like the tetracysteine tag, Glu^494^ is in a disordered and evolutionarily divergent region and is proximal to 2CARD (Fig. 1 and fig. S1). Thus, the incorporated tags are both solvent-exposed to facilitate labeling; they provide orthogonal chemical functionality; and their incorporation is unlikely to perturb RIG-I function.

### Creation and labeling of the RIG-I FRET reporter

**Fig. 2:**
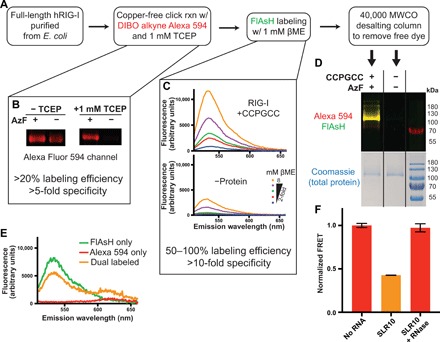
Dual-labeled RIG-I reports 2CARD ejection by change in FRET. (**A**) A schematic representation of the process used to dual-label RIG-I with donor and acceptor fluorophores. Critical features of this protocol are highlighted. (**B**) One micromolar TCEP is required to reduce nonspecific labeling of RIG-I by DIBO alkyne Alexa Fluor 594. Labeled protein was analyzed by SDS–polyacrylamide gel electrophoresis (PAGE), followed by fluorescent imaging (excitation/emission: 532 nm/LP575). In the absence of TCEP, negative control protein lacking AzF (−AzF) is nonspecifically labeled. (**C**) βME is required for efficient FlAsH labeling (top), but large amounts of βME cause nonspecific fluorescence by free FlAsH (bottom). One micromolar βME was identified as an ideal concentration that is sufficient for labeling without causing background fluorescence. Fluorescent emission spectra were measured after FlAsH excitation at 479 nm. (**D**) The optimized protocol produces specifically dual-labeled RIG-I. Tagged and untagged proteins were both used in the dual-labeling protocol and analyzed by fluorescent imaging of an SDS-PAGE gel (Alexa Fluor excitation/emission: 532 nm/LP575; FlAsH excitation/emission: 473 nm/530DF20). Only tagged protein was covalently modified, demonstrating the specificity of labeling. (**E**) Dual-labeled protein exhibits FRET. Fluorescent emission spectra were measured after FlAsH excitation at 479 nm, demonstrating acceptor emission at ~615 nm, significantly above bleed-through fluorescence from direct excitation/emission of individual fluorophores. (**F**) RNA causes a decrease in FRET corresponding to an ejection of the 2CARD domain. Treatment with benzonase digests the RNA and resets the 2CARD domain. Error bars correspond to the SEM across replicate samples.

We encountered several challenges in the creation of the dual-labeled FRET reporter that required innovative steps to overcome. The azide group of AzF is prone to reduction to a nonreactive amine, so we decreased protein expression time and omitted reducing agents during purification (*30*). In addition, we performed click labeling before FlAsH labeling because the FlAsH labeling reaction requires reducing agents (Fig. 2A). Traditional click labeling requires a copper catalyst that negatively affected RIG-I activity. However, a strained alkyne moiety, such as dibenzylcyclooctyne (DIBO), eliminates the need for copper in the click reaction. Several DIBO-modified fluorophores are commercially available, including Alexa Fluor 594, which has good spectral overlap with FlAsH. Copper-free click labeling with DIBO Alexa Fluor 594 was initially successful but was not specific for AzF-containing protein (Fig. 2B). We hypothesized that the nonspecific reaction was occurring via thiol-yne addition to cysteines, so we added a small amount of reducing agent and saw a marked increase in specificity without a significant reduction in labeling efficiency (Fig. 2B) (*31*, *32*).

FlAsH labeling was also inefficient before the addition of precise amounts of 2-mercaptoethanol (βME) (Fig. 2C). FlAsH has the unique feature of being quenched in solution when bound to ethanedithiol (EDT), significantly reducing background signal from free fluorophore (*33*). This quenching is alleviated when the tetracysteine tag displaces EDT. However, βME also displaces EDT and increases background signal from free FlAsH (Fig. 2C). Thus, precise concentrations of βME and EDT were required to achieve specific and efficient FlAsH labeling.

Optimized labeling reactions produced active, dual-labeled RIG-I that exhibits a specific FRET signal in the autoinhibited state. After dual-labeling RIG-I, excess free fluorophore was removed (Fig. 2A), and RNA binding activity was measured. Dual-labeled RIG-I binds RNA with a similar affinity to untagged wild-type protein (*K*_D,WT_ = 2.2 ± 0.2 nM and *K*_D,labeled_ = 1.2 ± 0.1 nM), suggesting that neither the tags nor fluorophores perturb RIG-I function (fig. S2A). SDS–polyacrylamide gel electrophoresis (PAGE) analysis demonstrated that dual labeling requires incorporation of the tetracysteine and AzF tags, allowing us to attribute any FRET signal to the specifically tagged locations (Fig. 2D). The fluorescent emission spectrum upon excitation of FlAsH (donor) revealed a peak corresponding to Alexa Fluor 594 (acceptor) emission (Fig. 2E). This Alexa Fluor 594 signal was substantially higher than that observed for singly labeled proteins, suggesting that it derives from FRET, rather than spectral bleed-through from direct excitation/emission of individual fluorophores. Unfortunately, some noncovalently bound Alexa Fluor 594 copurified with the dual-labeled protein, but controls using a protein with only the tetracysteine tag showed that the FRET signal does not derive from the copurified Alexa Fluor 594 (fig. S2, B and C). Thus, we observe a FRET signal for active, dual-labeled RIG-I that specifically corresponds to the distance between the 2CARD and hel2i domains.

### RNA binding ejects the 2CARD domain

RNA binding causes a reduction in FRET consistent with the ejection of 2CARD from the body of the protein (Fig. 2F). FRET was measured by quantifying the acceptor fluorophore emission upon excitation of the donor fluorophore. After correcting for bleed-through and loading, this FRET signal was normalized to maximum and minimum FRET states corresponding to the autoinhibited state and a denatured sample, respectively. Thus, a value of 1 corresponds to the high-FRET autoinhibited state, and a value of 0 corresponds to a low-FRET state where the fluorophores are as far apart as possible. When an optimal RIG-I RNA ligand (SLR10) was added, the FRET signal decreased significantly, suggesting an increase in distance between the 2CARD and hel2i domains (Fig. 2F), consistent with the exceptional potency of this ligand (*3*). RNA binding did not significantly affect the fluorescence intensity of individually labeled proteins, suggesting that the FRET change derives from an increase in distance between fluorophores, rather than an artifact of fluorescence quenching or enhancement (fig. S2D). The observed decrease in FRET occurred faster than could be detected with the current experimental setup (~5-s resolution), consistent with the on-rate (*t*_1/2_ ≈ 0.1 s) that would be predicted for a high-affinity RNA such as SLR10 [*K*_D_ ≈ 160 pM (*19*) and *k*_off_ = 0.028 s^−1^ (*18*)] at a concentration of 100 nM with 50 nM protein. Nuclease digestion of the RNA caused an increase in FRET, back to the original level, demonstrating that the FRET change is RNA dependent and reversible. This is the first time that RIG-I activation has been shown to be reversible and that the protein can “reset” by restoring 2CARD to its original position in the inactive state.

**Fig. 3:**
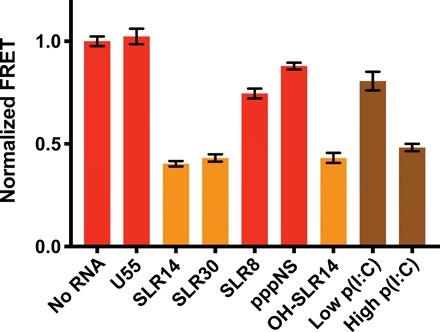
RNA binding ejects the 2CARD domain. Optimal RIG-I ligands containing 5′-triphosphorylated blunt duplex stems (SLR14 and SLR30) eject the 2CARD domain, whereas RNAs that do not bind (U55) do not eject the 2CARD domain. A duplex longer than 8 bp is required for efficient 2CARD ejection (SLR8). The 5′ triphosphate is not strictly required for 2CARD ejection (OH-SLR14), but internal duplexes do not eject the 2CARD domain {low polyinosinic:polycytidylic acid [poly(I:C)]}. Two molar equivalents of RNA were added to 50 nM protein to ensure saturation. Poly(I:C) was added at an equivalent concentration to SLR10 [low poly(I:C) = 0.79 ng/μl] or at 100 times the concentration [high poly(I:C) = 79 ng/μl)]. Error bars correspond to the SEM across replicate samples.

Next, we sought to determine the structural requirements for RNA-mediated 2CARD ejection. Other optimal RIG-I ligands (SLR14 and SLR30) eject 2CARD to similar extents, whereas RNAs that do not bind RIG-I (U55 and pppNS) do not eject 2CARD, further demonstrating the specificity of the assay (Fig. 3). SLR10, SLR14, and SLR30 are 5′-triphosphorylated blunt duplex RNA hairpins that contain 10, 14, and 30 base pairs (bp), respectively. A slightly shorter RNA hairpin, SLR8, binds with high affinity, but does not signal in cell culture, exemplifying the limitations of existing in vitro binding assays (*20*). SLR8 would be expected to partially clash with 2CARD, if bound in the same manner as longer RNAs. The FRET assay reveals that SLR8 inefficiently ejects 2CARD, suggesting the formation of a protein-RNA complex distinct from the structurally characterized conformations and providing a mechanistic explanation for the inability of this ligand to signal in cells. Thus, an RNA stem longer than 8 bp is required for 2CARD ejection, but additional increases beyond 10 bp do not affect 2CARD ejection.

We also tested a 5′-triphosphorylated RNA equal in length to SLR10 but lacking the duplex structure (pppNS). pppNS binds RIG-I weakly with a low-micromolar *K*_D_ and does not activate RIG-I in vivo (*3*, *19*). The FRET assay uses nanomolar concentrations of protein and RNA, meaning that pppNS would not be expected to bind, and we see that pppNS does not cause 2CARD ejection (Fig. 3). However, pppNS can cause 2CARD ejection at low-micromolar concentrations, consistent with its *K*_D_ (fig. S3). These results demonstrate the utility of the FRET assay over other assays that require higher concentrations of reagents. The FRET assay is uniquely able to differentiate between high- and low-affinity RIG-I ligands because of the low concentrations of reagents it requires. These observations further suggest that, above a certain RNA length, the structural requirements for 2CARD ejection are lax and that RIG-I activation in vivo is largely dictated by its relative affinity for a given RNA.

Last, we tested how changes to the 5′ end of the RNA affect 2CARD ejection. OH-SLR14 has the same blunt duplex as SLR14, but it has a 5′-OH in place of the triphosphate. This RNA has decreased affinity for RIG-I and does not activate RIG-I in vivo (*3*, *19*). However, RIG-I binds OH-SLR14 with moderate affinity (low-nanomolar *K*_D_), and OH-SLR14 can activate RIG-I mutants that are deficient in their ability to dissociate from RNA (*34*). We observe that OH-SLR14 ejects 2CARD when bound to RIG-I (Fig. 3). It is important to note that the concentrations of protein and RNA used in this assay are greater than the *K*_D_ for OH-SLR14, ensuring that RIG-I is fully bound to OH-SLR14. Thus, the 5′ triphosphate is not strictly required for 2CARD ejection, as long as RNA binding can still occur. Similarly, polyinosinic:polycytidylic acid [poly(I:C)] lacks a 5′ triphosphate and is capable of ejecting 2CARD (Fig. 3). However, by mass, more poly(I:C) than SLR10 is required to eject 2CARD, consistent with the fact that poly(I:C) has a lower concentration of blunt duplex ends than SLRs (Fig. 3) (*20*). This further suggests that the internal duplex binding sites present in poly(I:C) are not capable of binding RIG-I and ejecting 2CARD at the concentrations used in this assay. In summary, RNA duplexes greater than 8 bp are sufficient for 2CARD ejection, and additional structural features, such as a blunt terminus and 5′ triphosphate moiety, serve to increase binding affinity.

### ATP has little effect on 2CARD ejection

**Fig. 4:**
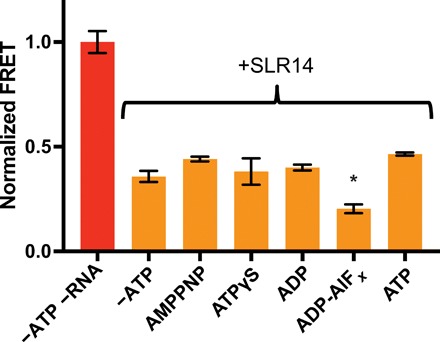
ATP analogs have little effect on the conformation of the 2CARD domain. RIG-I was mixed with RNA, followed by the indicated ATP analog. A one-way analysis of variance (ANOVA) identified an effect of ATP analog on 2CARD ejection (*F*_5,12_ = 9.362, *P* = 0.0008) and a Dunnett’s post hoc test identified adenosine diphosphate (ADP)–AlF*_x_* as the only sample significantly different from the +SLR14 sample that lacked ATP analog (*P = 0.0143). Error bars correspond to the SEM across replicate samples.

ATP binding also plays a role in RIG-I signaling; thus, we tested the effects of ATP and various analogs on the conformation of the 2CARD domain. The ability of RIG-I to bind ATP correlates with its ability to signal in cells (*18*). In addition, the inability of RIG-I to hydrolyze ATP leads to its overactivation (*5*, *22*). These observations led to the hypothesis that, following RNA binding, ATP binding promotes signaling by enhancing 2CARD ejection (*18*, *35*). However, we observed no major change in 2CARD ejection upon the addition of nonhydrolyzable ATP analogs (AMP-PNP and ATPγS), which should mimic an ATP-bound conformation of RIG-I (Fig. 4). Adenosine diphosphate (ADP) and ATP do not influence 2CARD ejection, but the transition-state analog ADP-AlF*_x_* appears to cause a subtle decrease in FRET. ATP analogs have similarly minor effects in the presence of SLR30 and the absence of RNA, further demonstrating that RNA length does not affect 2CARD ejection (fig. S4, A and B). After repeating the ADP-AlF*_x_* experiment, the decrease in FRET is significant but subtle (fig. S4C). The normalized FRET value with ADP-AlF*_x_* is greater than 0 (the level of denatured protein), suggesting that the FRET assay is still within its dynamic range. Therefore, ADP-AlF*_x_* causes a reproducible but small change in the position of the 2CARD domain.

## DISCUSSION

Here, we have used a novel assay to reveal key features about the interplay between RNA and ATP in RIG-I activation. First, we have provided important insight into how RIG-I avoids unwanted activation by self-RNA and how this might become misregulated in autoimmune diseases, such as Singleton-Merten syndrome (SMS) (*5*). We have shown that activation of RIG-I is not strictly dependent on the 5′ triphosphate moiety, suggesting that self-RNA is capable of activating RIG-I under misregulated conditions. Such misregulation could occur for a variety of reasons, including an increase in cellular RNA concentration or misregulation of RIG-I binding. For example, patients with SMS have mutations in RIG-I that block its ATPase activity, preventing dissociation of RIG-I from self-RNA (*5*, *18*, *22*, *36*). However, there was previously no direct evidence that this dissociation from RNA prevented signaling. Here, we provide the first direct evidence that the 2CARD signaling domain rapidly returns to its autoinhibited state after RNA dissociation, supporting the current model that SMS is caused by impaired ATP hydrolysis, impaired dissociation of RIG-I from self RNA, and, ultimately, impaired sequestration of the 2CARD domain (*18*, *22*, *36*).

The ability of 2CARD to reset after dissociation from RNA has implications for mechanisms of downstream signaling. One model for RIG-I signaling involves the formation of a homotetramer via the 2CARD domain (*37*). In the case of SLR14, this would require the simultaneous encounter of four individual protein-RNA complexes. This process would be extremely inefficient if RIG-I were also dissociating from RNA and reverting to its autoinhibited state, yet SLR14 is a potent RIG-I agonist in vivo (*3*). There is also evidence that ubiquitination of the 2CARD domain promotes RIG-I signaling, especially on short RNAs (*16*, *38*). Thus, it is tempting to speculate that ubiquitination and, perhaps, other modifications of the 2CARD domain prevent 2CARD reset, trapping RIG-I in an active state and facilitating the formation of a tetramer.

It is particularly significant that RNA alone is sufficient to cause 2CARD ejection and that presentation of 2CARD does not require ATP. These observations are important in light of the many different functions that have been ascribed to ATP binding and hydrolysis by RIG-I (*18*, *23*–*25*), and they underscore the primary role of RNA binding during activation of the RIG-I sensor. They explain the observation that short (10 to 14 bp) triphosphorylated RNA ligands are particularly potent RIG-I agonists and that these small RNAs can stimulate signaling even by ATPase-deficient RIG-I mutants (*18*). However, they do not explain the fact that, although RIG-I can bind tightly to the terminus of any triphosphorylated RNA duplex, regardless of length (*20*), it can only signal from longer double-stranded RNAs (dsRNAs) if ATP-binding activity is maintained (*18*). One explanation is that the 2CARD ejection caused by RNA alone might be insufficient for signaling. In the presence of ADP-AlF*_x_*, there is a small but observable decrease in FRET signal, and it is known that ADP-AlF*_x_* can mimic a transient transition state during ATP hydrolysis that may somehow populate a conformation that assists in CARD presentation. However, the dispensability of ATP hydrolysis for RIG-I signaling in cells undercuts this model (*18*, *22*). An alternative explanation is that RNA alone is sufficient to eject 2CARD and activate RIG-I in a pure system and on short RNAs in cells but that longer RNAs such as SLR30 are coated by cellular factors that repress signaling by ATPase-deficient RIG-I mutants. In this case, the ATPase activity of RIG-I may have a second function, enabling RIG-I to displace competing cellular factors to signal from the dsRNA terminus (*39*). Shorter RIG-I agonists, such as SLR10 and SLR14, are still able to induce cell signaling in ATPase-deficient RIG-I mutants because they may lack binding sites for additional cellular factors and/or the tightly bound RIG-I molecule that caps the short duplex terminus does not leave any room for competing factors to bind. On the basis of the available data from all laboratories, we favor a model in which ATP plays no substantive role in 2CARD presentation or for the conformational changes needed for ejecting 2CARD from its Hel2i-binding site, but that ATP may be required for displacement of competing proteins and (as has now been demonstrated by multiple laboratories) for proofreading of inappropriate RIG-I targets such as host RNA (*18*, *22*, *23*, *26*, *34*, *35*).

That said, if RIG-I is capable of displacing proteins from long RNA targets, it is expected to proceed through the winch-like mechanism that is commonly observed for protein displacement by DEAD-box proteins (*40*), as RIG-I and related ATPases are structurally and phylogenetically related to DEAD-box proteins (*41*) rather than processive viral helicases (such as NPH-II) (*42*). Evolution of the pincer domain in RIG-I–like proteins resulted in a conformational alteration that prevents translocation via the mechanism established for SF2 proteins such as NS3 (*43*), perhaps explaining slow rates of apparent RIG-I motion on RNA (*23*) and suggesting a nonprocessive mechanism much like that reported for related DEAD-box RNPases (*40*).

Recent publications have also reported 2CARD ejection by RNA and ATP analogs using HDX and SAXS, but the FRET assay adds important quantitative information that leads us to a slightly different conclusion (*26*–*28*). Using HDX, researchers observed an increase in 2CARD solvent exposure upon binding RNA (*28*). Like the FRET assay, HDX reports an additional increase in solvent exposure upon the addition of ADP-AlF*_x_* (*28*). However, the extent of the conformational change upon the addition of ADP-AlF*_x_* was unclear, and it is curious that no other ATP analog showed this effect. On the basis of these data, the authors concluded that ATP plays a significant role in RIG-I activation (*28*). SAXS, on the other hand, reports a quantitative increase in molecular size upon RNA and ADP-AlF*_x_* binding, but the domains responsible for this increase are ambiguous, and again, the effect was only observed with ADP-AlF*_x_* (*27*). These results led to the conclusion that ATP is important for CARD ejection, but the conformational changes were small and cannot be unambiguously attributed to motion of the 2CARD domain. FRET provides a more specific and quantitative metric for 2CARD ejection. This new metric reveals substantial 2CARD ejection upon RNA binding and a relatively small change caused by ADP-AlF*_x_*. In combination with the body of work described above, RNA alone appears sufficient for 2CARD ejection and activation of RIG-I.

The FRET assay has certain technical advantages over other 2CARD ejection assays that aid interpretation of results and will benefit future studies. The FRET assay uses low- to mid-nanomolar concentrations of protein and RNA, whereas structural studies such as HDX and SAXS use much higher concentrations in the low- to mid-micromolar range. The lower concentrations required for FRET are more biologically relevant and less prone to artifacts, such as homodimerization of RNA hairpins and protein multimerization. The low concentrations made it possible to differentiate between high- and low-affinity binding events, such as the difference between SLR and pppNS binding. The FRET assay can also be adapted for future studies of RIG-I that would be intractable with HDX or SAXS. First, FRET is more accessible than HDX and SAXS as it requires only a fluorometer, rather than a mass spectrometer or x-ray source. Second, the readout for the FRET assay is rapid and facile, which allows for use in screening and kinetic experiments. Last, this FRET construct could be used in single-molecule experiments to identify transient or lowly populated states involved in RIG-I activation (*44*).

Unlike HDX and SAXS, FRET requires careful design and validation of a construct, which we have performed here. We placed small nonperturbing fluorophores at locations as proximal as possible in the high-FRET state. There are relatively few methods able to attach small fluorophores at specific internal protein locations, and these methods are further restricted for large and unstable proteins such as RIG-I. Several labeling strategies require pH, temperatures, or reagents that adversely affected RIG-I activity. FlAsH and AzF were ultimately successful because they require mild conditions and commercially available reagents. Here, we have optimized these strategies for use together, creating a dual-labeling strategy that is applicable to other large proteins.

We have created and used a novel method to address the unmet need for a biochemical assay that measures RIG-I activation in vitro. Previous biochemical studies of RIG-I have focused on RNA binding and ATP hydrolysis activities, but these activities do not necessarily correlate with RIG-I activation. There are many steps between RNA binding/ATP hydrolysis and signaling in a cell; thus, it is expected that the correlations between these activities are imperfect. Our 2CARD ejection assay measures a structural change more closely related to signaling and, therefore, more closely reports activation of RIG-I.Our FRET assay can be adapted for a variety of experiments on RIG-I, and similar methodology could be applied to homologous proteins, such as MDA5. In addition, we have provided a roadmap for the site-specific dual labeling of large proteins that will facilitate similar studies in many biological systems.

## MATERIALS AND METHODS

### Cloning and protein construct creation

Full-length human RIG-I was previously cloned into a pET SUMO expression vector (*19*). This construct contains an N-terminal 6-His tag, followed by a SUMO tag and the complete human RIG-I protein sequence. A serine residue was also added between the SUMO tag and RIG-I to ensure efficient tag cleavage.

In this study, we added the amino acid sequence MPCCPGCCGS following RIG-I residue 190. This sequence was added using Q5 mutagenesis (New England Biolabs) according to the manufacturer’s instructions. AzF was incorporated by amber stop codon suppression. Thus, the wild-type amino acid Glu^494^ was mutated to an amber stop codon (UAG) by the QuikChange Site-Directed Mutagenesis Kit (Agilent) according to the manufacturer’s recommendations.

### RIG-I expression and purification

RIG-I was expressed and purified essentially as previously described (*19*). Plasmid was transformed into Rosetta (DE3) competent cells (Novagen). Cells were grown at 37°C in Luria broth (Research Products International) to an optical density at 600 nm (OD_600_) of 0.6. Upon reaching 0.6, cells were cold-shocked on ice for 20 min and then returned to a 16°C incubator. Protein expression was induced with 0.3 mM isopropyl-β-d-thiogalactopyranoside (IPTG), and cells were grown for 16 to 24 hours.

Cells were harvested by centrifugation and resuspended in 10-ml lysis buffer per gram of cell paste [25 mM K-Hepes, 300 mM NaCl, 5 mM βME, 5 mM imidazole, 10% glycerol (pH 8.0)]. One EDTA-free protease inhibitor tablet (Roche) was added, and cells were lysed by passing three to five times through a microfluidizer. Lysate was clarified by centrifugation at 15,000*g* for 30 min. Clarified lysate was batch-incubated with Ni-NTA agarose beads for 1 hour, washed with 5 column volumes wash buffer [25 mM K-Hepes, 1 M NaCl, 30 mM imidazole, and 10% glycerol (pH 8.0)], and eluted [25 mM K-Hepes, 300 mM NaCl, 5 mM βME, 250 mM imidazole, and 10% glycerol (pH 8.0)]. Eluant was diluted 1:1 with elution buffer lacking imidazole and incubated with Ulp1 SUMO protease overnight at 4°C. Cleaved protein was further diluted 1:1 with 25 mM K-Hepes, 5 mM βME, and 10% glycerol (pH 8.0) and loaded onto a HiTrap Heparin HP column (GE Healthcare). The column was washed with 25 mM K-Hepes, 150 mM NaCl, 10% glycerol, and 5 mM βME (pH 8.0) and eluted with 25 mM K-Hepes, 650 mM NaCl, 10% glycerol, and 5 mM βME (pH 8.0). Eluent from the heparin column was concentrated and further purified on a HiLoad Superdex 200-pg column (GE Healthcare) equilibrated in 25 mM K-Hepes, 150 mM NaCl, and 5% glycerol (pH 7.5). Monomeric protein was collected, concentrated, flash-frozen in liquid nitrogen, and stored at −80°C.

RIG-I containing AzF was expressed and purified in a similar manner with several alterations. BL21(DE3) *Escherichia coli* were transformed with 200 ng of pSUMO–RIG-I containing an amber stop codon and 200 ng of pEVOL-pAzF. pEVOL-pAzF was a gift from P. Schultz (Addgene plasmid #31186; http://n2t.net/addgene:31186; RRID:Addgene_31186) (*45*). Cells were grown to an OD_600_ of 0.4, the temperature was decreased to 16°C, and 4-azido-l-phenylaline (0.25 g/liter) was added. l-Arabinose (0.02%) was added after 5 min. Cells were further grown to an OD_600_ of 0.6 when 0.3 mM IPTG was added, and cells were grown for 8 to 9 hours. Cells were harvested, and protein was purified as described for wild-type RIG-I except that exposure to light was limited and βME was omitted to prevent reduction of the azide.

### RNA preparation

Triphosphorylated SLR10, SLR14, and pppNS were synthesized on a MerMade synthesizer (BioAutomation) and deprotected using ammonium hydroxide as previously described (*46*, *47*). U55 and 5′-OH RNAs were synthesized using standard phosphoramidite chemistry and deprotected as previously described (*48*).

SLR30 was transcribed in vitro from a double-stranded synthetic DNA template (Integrated DNA Technologies) containing 2′-OMe modifications on the last two nucleotides of the template strand. One microgram of annealed template was used in a 100-μl transcription reaction containing 5 mM each ribonucleoside triphosphate, 22 mM magnesium chloride, 40 mM tris-HCl (pH 8.0), 2 mM spermidine, 10 mM dithiothreitol (DTT), 0.01% Triton X-100, 40 U of ribonuclease (RNase) inhibitor (New England Biolabs), and 5 μl of T7 RNA polymerase. Transcription reactions were incubated at 37°C for 2 to 12 hours.

All RNA was gel-purified by denaturing gel electrophoresis. Bands were excised, and RNA was extracted by crush soak in 10 mM Mops (pH 6.0), 300 mM NaCl, and 1 mM disodium-EDTA. RNA was ethanol-precipitated, washed once with 70% ethanol, and dissolved in water. RNA purity was confirmed by mass spectrometry (Novatia LLC). Low–molecular weight poly(I:C) was purchased from Invivogen and used without further purification.

### Fluorophore conjugation

RIG-I containing the tetracysteine tag and E494AzF mutation was diluted to 25 μM in 25 mM K-Hepes and 150 mM NaCl (pH 7.5). Unless otherwise indicated, 1 mM TCEP-HCl (pH 7.5) was added, immediately followed by 50 μM Click-IT Alexa Fluor 594 DIBO Alkyne (Thermo Fisher Scientific) dissolved in dimethyl sulfoxide (DMSO). The final DMSO concentration in the click labeling reaction was less than 5%. The reaction was incubated at 4°C overnight.

FlAsH labeling was performed after overnight click labeling. One millimolar TCEP-HCl, 1 mM βME, 1 mM EDT, and 70 μM FlAsH-EDT_2_ were added, and the reaction was incubated for 2 hours at 4°C. Excess free fluorophore was removed using a 40,000 molecular weight cutoff Zeba desalting column (Thermo Fisher Scientific) equilibrated with 25 mM K-Hepes, 150 mM NaCl, and 1 mM TCEP-HCl (pH 7.5).

After labeling and desalting, concentrations of protein and fluorophores were calculated by spectrophotometry using the extinction coefficients of ε_515_ = 41,000 M^−1^ cm^−1^ and ε_280_ = 4100 M^−1^ cm^−1^ for FlAsH, ε_590_ = 73,000 M^−1^ cm^−1^ and ε_280_ = 40,900 M^−1^ cm^−1^ for Alexa Fluor 594, and ε_280_ = 99,700 M^−1^ cm^−1^ for RIG-I. Samples were also analyzed by SDS-PAGE using a 12% acrylamide tris-glycine gel and loading dye that contained 50 mM TCEP, rather than βME, to prevent dissociation of the FlAsH fluorophore. Fluorescent imaging of the gels was performed on a Typhoon FLA 9500 biomolecular imager (GE Healthcare), followed by Coomassie staining. The intensities of bands were quantified manually in ImageQuant (GE Healthcare), and labeling specificity was calculated as the ratio of fluorescence intensity for tagged protein over untagged protein. Labeling efficiency was calculated as the concentration of fluorophore divided by the concentration of protein, as measured by spectrophotometry. Copurified free Alexa Fluor 594 was corrected for by measuring the fraction of total fluorescent signal on the SDS-PAGE gel derived from the protein band.

### RIG-I CARD ejection assay

Dual-labeled RIG-I was mixed with RNA in a black 96-well nonbinding plate (Corning). ATP analogs were added, as indicated, and reactions were equilibrated briefly on ice. ADP, ATP, and AMP-PNP were purchased from Sigma-Aldrich, and ATPγS was purchased from Cayman Chemical. A 3× stock of ADP-AlF*_x_* was created by mixing 6 mM ADP, 30 mM NaF, and 6 mM AlCl_3_, followed by 0.2-μm filtration. Final reactions contained 25 mM K-Hepes (pH 7.5), 75 mM NaCl, 75 mM KCl, bovine serum albumin (0.1 mg/ml), 0.3 mM TCEP-HCl, 50 nM RIG-I, 100 nM RNA, and 2 mM MgCl_2_/ATP analog. Reactions were set up at least in triplicate, and measurements were collected 5, 15, 30, 45, and 60 min after mixing. We observed no change in fluorescence over time; thus, readings for individual wells were averaged across time points. Reported error corresponds to the SE between replicate wells. RNase digestion of RNA was achieved by adding 500 U of benzonase (EMD Millipore) and 2 mM MgCl_2_, followed by incubation on ice for 30 min. Fluorescence was measured using a BioTek Synergy H1 plate reader with excitation/emission wavelengths of 479/615 nm (“FRET signal”), 479/532 nm (“donor signal”), and 580/615 nm (“acceptor signal”).

Proteins labeled with donor only or acceptor only were used to calculate the contribution of bleed-through to the FRET signal. Protein labeled with donor only had a bleed-through FRET signal that was 2.5% of the donor signal. Protein labeled with acceptor only had a bleed-through FRET signal that was 3.2% of the acceptor signal. Using these calibration values, a “subtracted FRET signal” was calculated using Eq. 1

Subtracted FRET=FRET signal−0.025*donor signal−0.032*acceptor signal (1)

Variations in loading were accounted for by normalizing this subtracted FRET signal to the acceptor signal according to Eq. 2

$$\text{Relative FRET}=\frac{\text{subtracted FRET}}{\text{acceptor signal}}$$ (2)

Last, the “relative FRET” value for each sample was normalized to a sample without RNA (maximal FRET) and a sample treated with 6 M urea (minimal FRET) to calculate a “normalized FRET” value according to Eq. 3

$$\text{Normalized FRET}=\frac{{\text{sample}}_{\text{relative FRET}}-{\text{urea}}_{\text{relative FRET}}}{\text{no}\;{\text{RNA}}_{\text{relative FRET}}-{\text{urea}}_{\text{relative FRET}}}$$ (3)

### Fluorescence anisotropy binding assay

Binding experiments were performed as described previously (*18*). The RNA used in these experiments consists of OH-SLR10 with Alexa Fluor 488 attached to an amine-modified uracil in the tetraloop (table S1). RNA was diluted in binding buffer [25 mM Mops (pH 7.4), 150 mM NaCl, 5 mM DTT, 2 mM MgCl, and 0.01% Triton X-100] to a concentration of 500 pM. RIG-I protein was serially diluted in binding buffer and mixed 1:1 with RNA in a 384-well nonbinding plate (Corning). Volumes were at least 20 μl. Plates were covered and incubated on ice for at least 2 hours until reactions reached equilibrium. Fluorescence polarization was measured using a Synergy H1 plate reader (BioTek). Samples were excited using a bandpass filter at 485/20 nm, and fluorescence emission was measured using a bandpass filter at 528/20 nm. Polarization was calculated using Eq. 4

$$\text{Polarization}=\frac{I_{\text{II}}-G*I_{⊥}}{I_{\text{II}}+G*I_{⊥}}$$ (4)

where *I*_II_ is the intensity of light parallel to the excitation plane, *I*_⊥_ is the intensity of light perpendicular to the excitation plane, and *G* is a correction factor empirically determined to be 0.87.

Protein dilutions were performed in triplicate, and averages were plotted against protein concentration. These data were fit to Eq. 4 using GraphPad Prism to calculate the dissociation constant

$$y=y_{0}+\frac{y_{\text{max}}*x}{K_{D}+x}$$ (5)

where *y* is the polarization value, *y*_0_ is the polarization value without protein, *y*_max_ is the polarization value at saturating RIG-I concentration, *K*_D_ is dissociation constant, and *x* is the concentration of RIG-I.

## Supplementary Material

http://advances.sciencemag.org/cgi/content/full/5/10/eaax3641/DC1

Download PDF

RNA binding activates RIG-I by releasing an autorepresed signaling domain

## SUPPLEMENTARY MATERIALS

Supplementary material for this article is available at http://advances.sciencemag.org/cgi/content/full/5/10/eaax3641/DC1 Fig. S1. Tags were placed in locations designed to facilitate labeling and avoid perturbation of RIG-I function. Fig. S2. Controls demonstrate that dual labeling of RIG-I does not perturb its function, and FRET reports the distance between hel2i and 2CARD. Fig. S3. pppNS ejects 2CARD at high concentrations. Fig. S4. ADP-AlF*_x_* subtly but reproducibly increases 2CARD ejection. Table S1. RNA sequences used in this study. References (*50*, *51*)
