# Supplementary material for: RNA binding activates RIG-I by releasing an autorepressed signaling domain
Source: Sci Adv. 2019 Oct 2;5(10):eaax3641. doi: 10.1126/sciadv.aax3641 (PMC6774723; doi:10.1126/sciadv.aax3641)
Supplement: http://advances.sciencemag.org/cgi/content/full/5/10/eaax3641/DC1 [file supp_5_10_eaax3641__index.html]

Science Advances | Science AdvancesAAASSearchScience AdvancesMenu

## Supplementary Materials

**This PDF file includes:**

- Fig. S1. Tags were placed in locations designed to facilitate labeling and avoid perturbation of RIG-I function.
- Fig. S2. Controls demonstrate that dual labeling of RIG-I does not perturb its function, and FRET reports the distance between hel2i and 2CARD.
- Fig. S3. pppNS ejects 2CARD at high concentrations.
- Fig. S4. ADP-AlF*x* subtly but reproducibly increases 2CARD ejection.
- Table S1. RNA sequences used in this study.
- References (*50*, *51*)

Download PDF

**Files in this Data Supplement:**

- Adobe PDF - aax3641\_SM.pdf
