# Supplementary material for: RNA binding activates RIG-I by releasing an autorepressed signaling domain
Source: Sci Adv. 2019 Oct 2;5(10):eaax3641. doi: 10.1126/sciadv.aax3641 (PMC6774723; doi:10.1126/sciadv.aax3641)
Supplement: Download PDF [file aax3641_SM.pdf]

Science Advances
AAAS

advances.sciencemag.org/cgi/content/full/5/10/eaax3641/DC1

Supplementary Materials for

RNA binding activates RIG-I by releasing an autorepressed signaling domain

T. H. Dickey, B. Song, A. M. Pyle*

*Corresponding author. Email: anna.pyle@yale.edu

Published 2 October 2019, *Sci. Adv.* **5**, eaax3641 (2019)
DOI: 10.1126/sciadv.aax3641

This PDF file includes:

Fig. S1. Tags were placed in locations designed to facilitate labeling and avoid perturbation of RIG-I function.
Fig. S2. Controls demonstrate that dual labeling of RIG-I does not perturb its function, and FRET reports the distance between hel2i and 2CARD.
Fig. S3. pppNS ejects 2CARD at high concentrations.
Fig. S4. ADP-AlF$_x$ subtly but reproducibly increases 2CARD ejection.
Table S1. RNA sequences used in this study.
References (*50*, *51*)

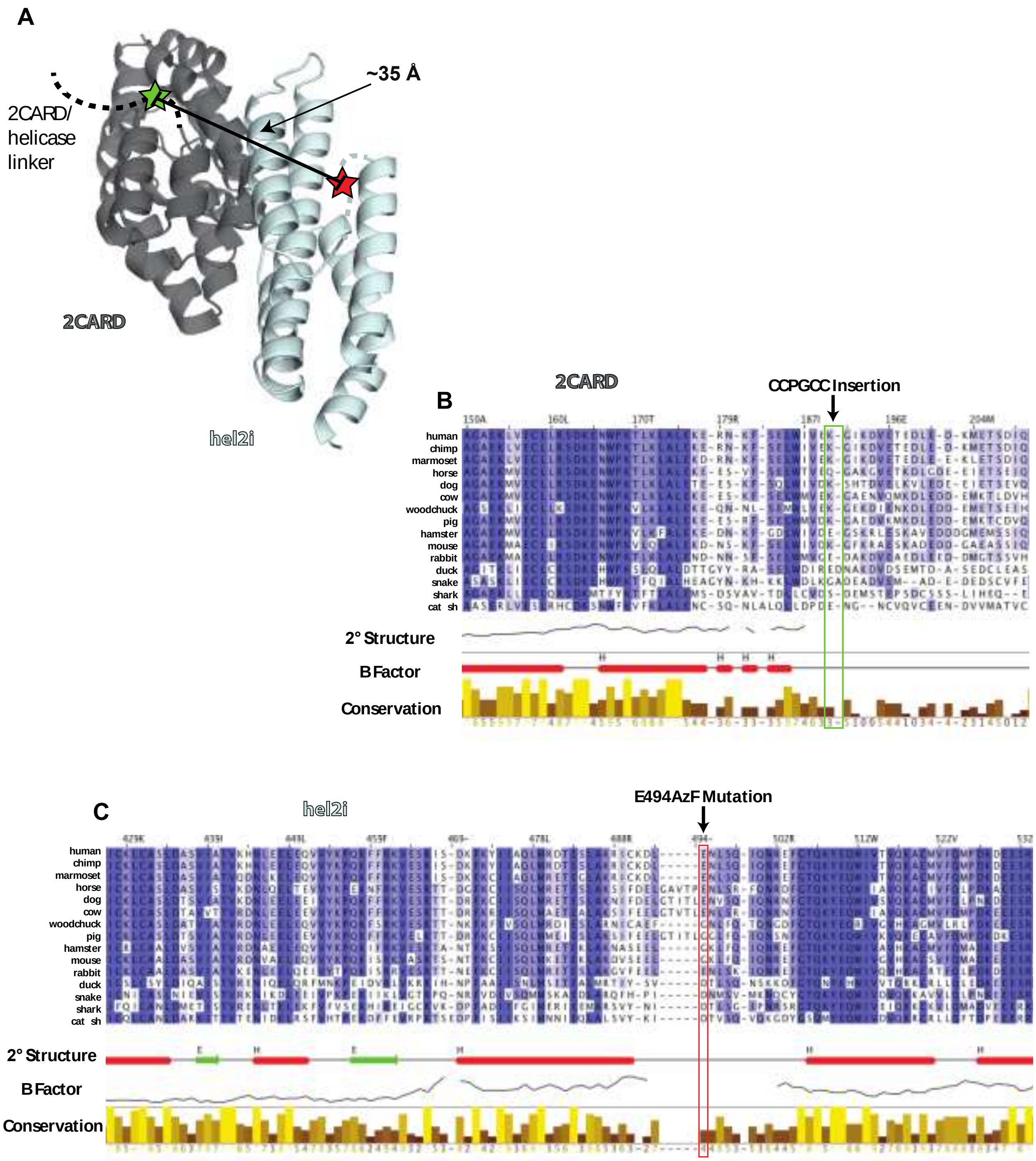

Fig. S1. Tags were placed in locations designed to facilitate labeling and avoid perturbation of RIG-I function. A) Tags were placed in regions of the 2CARD and hel2i domains that are disordered in the apo RIG-I structure (PDB: 4A2W) and predicted to be proximal. The distance between tags was estimated by identifying structures of isolated RIG-I domains (PDB:3ZD6 and PDB:2LWD) that modeled the amino acids at the locations of the tags. These structures were aligned to the structure of apo RIG-I and the distance between alpha carbons of the appropriate residues (E189 and E494 + 3.5 Å for the distance between amino acids 189 and 190) is reported. **B** and **C**) Tags were placed at evolutionarily divergent regions of RIG-I. Select sequences were aligned using the T-Coffee method in Jalview (*50*, *51*). Coloring indicates the level of conservation and secondary structures and B factors derive from the apo RIG-I structure.

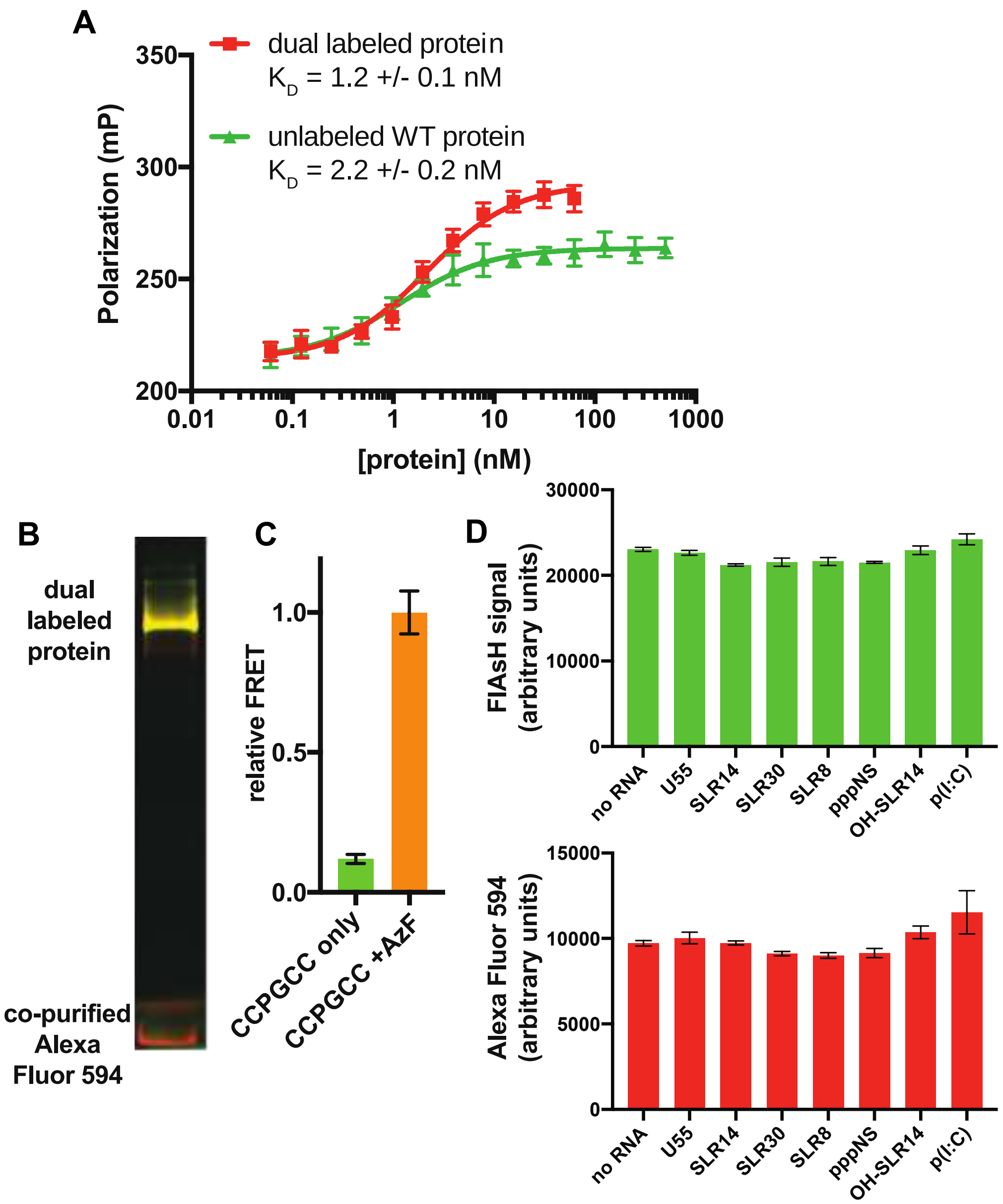

Fig. S2. Controls demonstrate that dual labeling of RIG-I does not perturb its function, and FRET reports the distance between hel2i and 2CARD. A) Dual-labeled RIG-I binds RNA with a similar affinity to un-tagged RIG-I. RNA binding activity was measured by fluorescence anisotropy using OH-SLR10 RNA. **B**) Free Alexa Fluor 594 co-purifies with dual-labeled RIG-I. Dual-labeled protein was purified by desalting column and analyzed by SDS-PAGE, revealing the presence of Alexa Fluor 594 that is not covalently bound to RIG-I. **C**) Non-covalently bound Alexa Fluor 594 does not contribute to the FRET signal. Without the AzF tag, Alexa Fluor is not covalently bound and exhibits little to no FRET signal. **D**) Changes in FRET are not due to artifacts of fluorescent quenching or enhancement upon RNA binding. Single-labeled proteins exhibit no significant change in fluorescence upon binding RNA, suggesting that FRET changes reflect a change in distance between fluorophores.

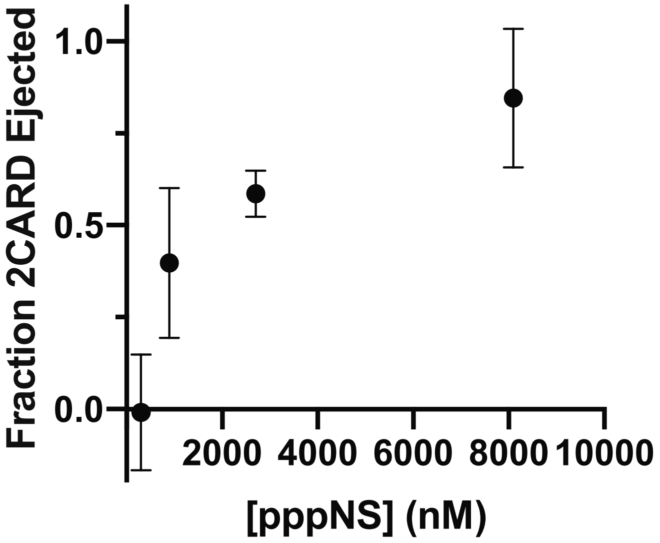

Fig. S3. pppNS ejects 2CARD at high concentrations. pppNS ejects 2CARD when bound at very high concentrations. This suggests that the structural requirements for 2CARD ejection are lax and that low concentrations of protein/RNA are required to discriminate between low- and high-quality RIG-I ligands. The fraction of 2CARD ejected was calculated by dividing the normalized FRET signal for each pppNS data point by a fully-ejected control sample bound to SLR14.

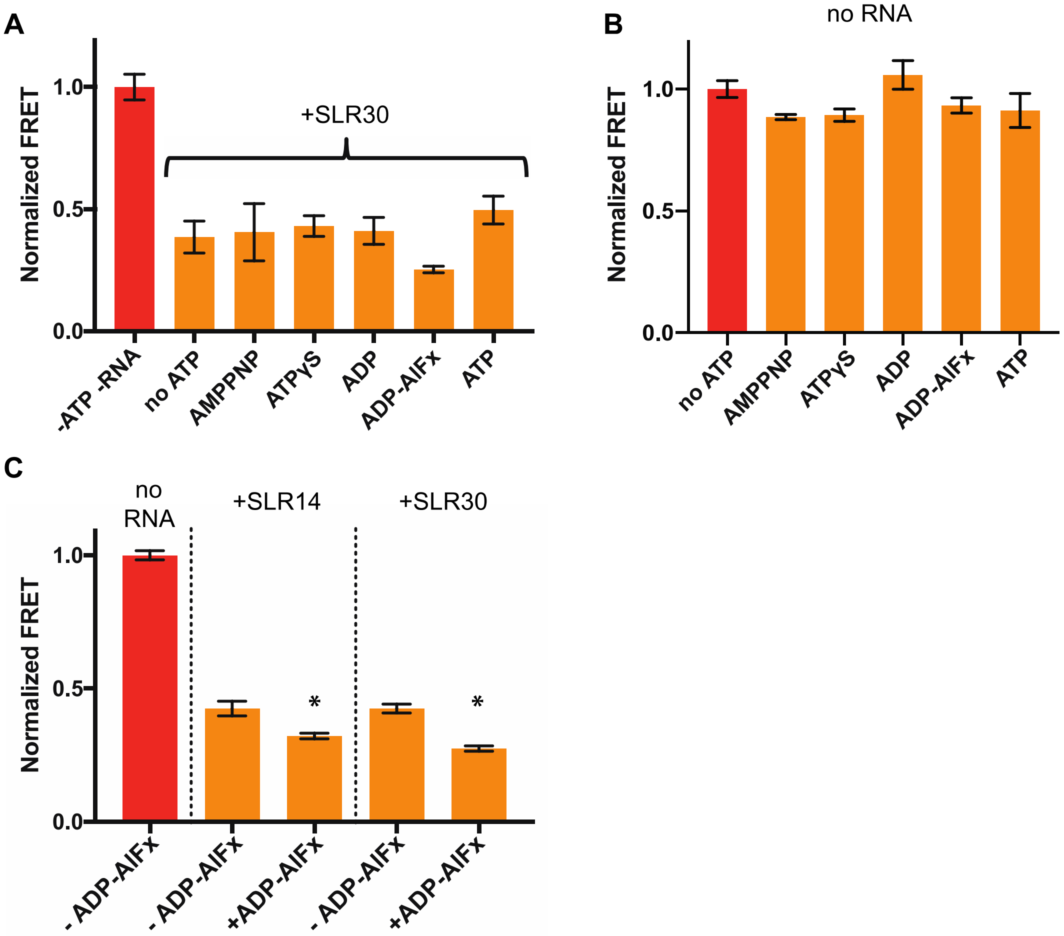

Fig. S4. ADP-AlF*x* subtly but reproducibly increases 2CARD ejection. A) Like SLR14, most ATP analogs have little effect on RIG-I bound to SLR30 **B**) and in the absence of RNA. **C**) Re-measuring the effect of ADP-AlF_x with a greater number of replicates reveals a significant decrease in FRET for RIG-I bound to both SLR14 (unpaired t-test; $t(10)=3.516$, * = $p = 0.0056$) and SLR30 (unpaired t-test; $t(10)=7.685$, * = $p = <0.0001$).

Table S1. RNA sequences used in this study.

SLR10	5′ ***ppp***-GGACGUACGU U<br>U<br>C<br>3′ OH-CCUGCAUGCA G
OH-SLR14	5′ OH-GGAUCGAUCGAUCG U<br>U<br>C<br>3′ OH-CCUAGCUAGCUAGC G
SLR14	5′ ***ppp***-GGAUCGAUCGAUCG U<br>U<br>C<br>3′ OH-CCUAGCUAGCUAGC G
SLR30	5′ ***ppp***-GGAUCGAUCGAUCGAUCGGCAUCGAUCGGC U<br>U<br>C<br>3′ OH-CCUAGCUAGCUAGCUAGCCGUAGCUAGCCG G
SLR8	5′ ***ppp***-GGCGCGGG U<br>U<br>C<br>3′ OH-CCGCGCCC G
pppNS	5′ ***ppp***-GAAGCAAUCUCCACUUACUAGAAA-OH3′
U55	5′ OH-U_{55}-OH 3′
OH-SLR10-AF488	5′ OH-GGACGUACGU U<br>U*AF488<br>C<br>3′ OH-CCUGCAUGCA G
